# Acute fibrinous and organizing pfneumonia: two case reports and literature review

**DOI:** 10.1186/s13000-021-01155-7

**Published:** 2021-10-10

**Authors:** Haihong Chen, Yukun Kuang, Xinyan Huang, Ziyin Ye, Yangli Liu, Canmao Xie, Ke-Jing Tang

**Affiliations:** 1 Division of Pulmonary and Critical Care Medicine, the First Affiliated Hospital of Sun Yat-sen University, Institute of Pulmonary Diseases, Sun Yat-sen University, Province Guangdong 510080 Guangzhou, People’s Republic of China; 2 Department of Pathology, the First Affiliated Hospital of Sun Yat-sen University, Province Guangdong, Guangzhou, People’s Republic of China

**Keywords:** Acute fibrinous and organizing pneumonia, Clinical characteristics, Biopsy, Pathology, Treatment

## Abstract

**Background:**

Acute fibrinous and organizing pneumonia (AFOP) is a rare histologic interstitial pneumonia pattern characterized by the intra-alveolar fibrin deposition and organizing pneumonia. Its clinical characteristics are still not well known and there is no consensus on treatment yet.

**Case presentation:**

We report two female cases in their fifties diagnosed with AFOP confirmed by a second lung biopsy. Case 1 was idiopathic AFOP with manifestation of 6-week fever, dyspnea, and cough, while case 2 was secondary to systemic lupus erythematosus and fever was the major symptom. Their chest CT scans revealed bilateral multiple consolidations, predominantly in the lower lobes. Both cases were initially diagnosed with pneumonia, but did not improve after treatment with broad-spectrum antibiotics. In both cases, transbronchial biopsy and bronchoalveolar lavage fluid examination were inconclusive and the pathological diagnosis was confirmed by percutaneous lung biopsy. Both patients had a good clinical response to prednisone.

**Conclusions:**

We report two rare AFOP cases to highlight the importance of awareness of this disease. We further perform the most comprehensive review to date in AFOP, including 150 patients since 2002. Consolidation was the most common imaging pattern, followed by ground-glass opacity and nodules. A lung biopsy is required for a definitive diagnosis. Corticosteroids is recommended as the most effective therapy, but treatment options should depend on the etiology and disease severity.

## Background

Acute fibrinous and organizing pneumonia (AFOP), first described in 2002 by Beasley [1], is a rare histologic interstitial pneumonia pattern characterized by the intra-alveolar fibrin deposition and organizing pneumonia [2]deposition and organizing.

AFOP has been frequently misdiagnosed due to inadequate knowledge of its pathology. Its clinical features are still not well known and there is no consensus on treatment yet. Clinical outcomes vary considerably according to its diversities, but most cases have ended up with poor prognosis.

Here we present two cases with a pathologic diagnosis of AFOP at our tertiary hospital in China. Furthermore, we describe the most comprehensive literate review of the AFOP, to provide further detail of this disease for physicinas.

## Case presentation

### Case 1

**Fig. 1:**
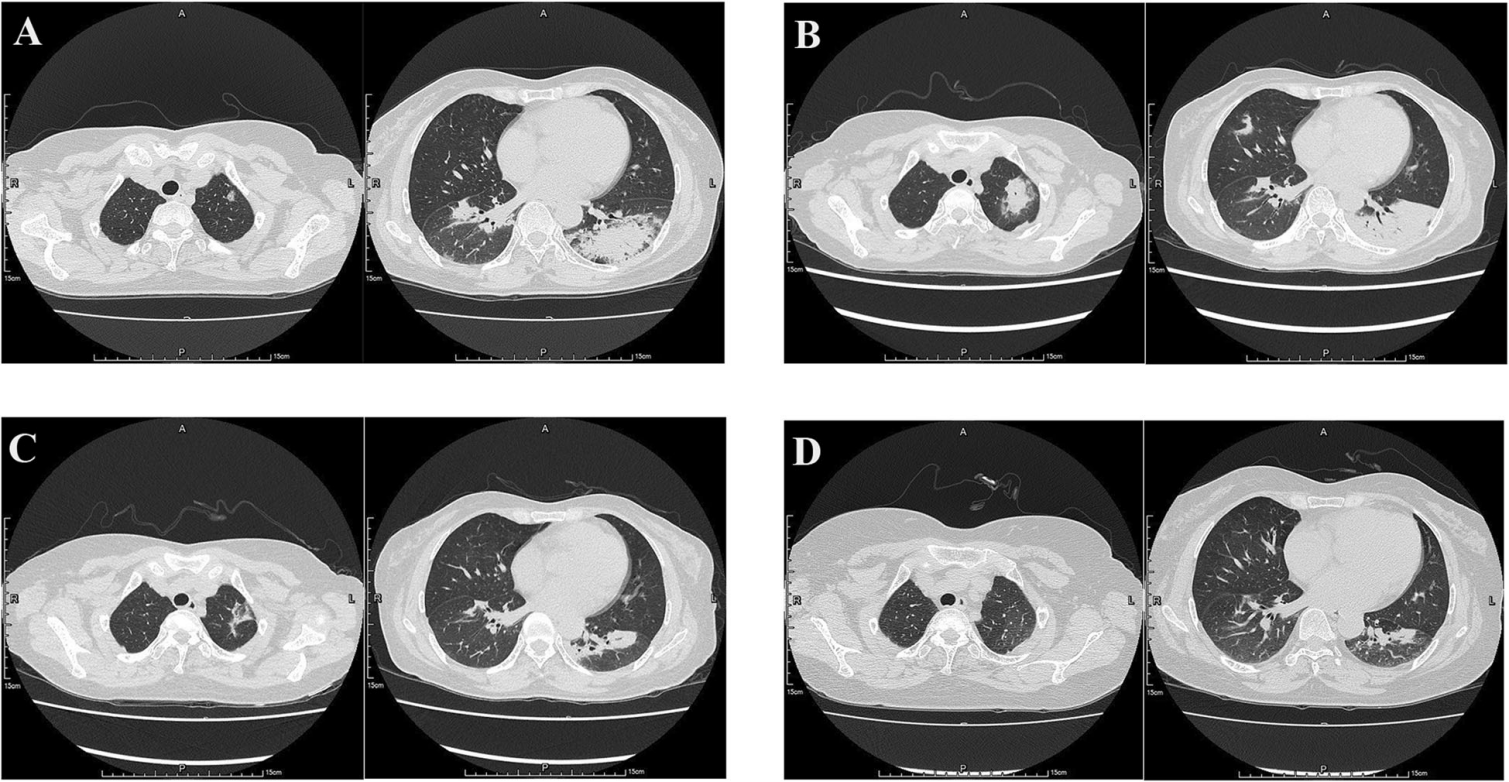
The initial and follow-up CT images of case 1. **A** CT of one day before admission showed bilateral consolidations, predominantly in both lower lobes, with basal and subpleural distribution, and patchy-like ground-glass opacity in the left upper lobe. **B** CT images on day 18th of admission showed lesions progression. **C** CT images of follow-up at the 12th day of the steroid treatment and **D** CT of follow-up at 3rd month after discharge showed lesions absorption

A 53-year-old female was transferred to our department with a 6-week history of dyspnea and cough with sputum in December 2018. She also complained of intermittent fever, mild chest pain, fatigue, appetite loss and night sweats. At a local hospital, her chest CT scan demonstrated multiple consolidations bilaterally, predominantly in both lower lobes, with mildly enlarged mediastinal lymph nodes. She was treated by ceftriaxone and levofloxacin, but her symptoms did not improve. CT scans (Fig. 1A) performed prior to hospitalization revealed enlarging bilateral lung lesions. She had past medical history of uterine fibroids, beta thalassemia (heterozygosity) and iron deficiency anemia.

Laboratory data showed normal white blood cell count(7.48*10^9/L)with elevated serum C-reactive protein (63.10 mg/L) and erythrocyte sedimentation rate (110mm/h). Serum procalcitonin (0.06ng/ml), 1-3-β-D Glucan (<37.5pg/ml) and Galactomannan measurement (0.1) were within normal range, and interferon release test for tuberculosis was negative. Blood and sputum cultures were negative. Tumor markers, including CEA, CA125, CA199, AFP and serological investigations for connective tissue disease, such as anti-nuclear antibodies, anti-dsDNA antibody, anti-neutrophil cytoplasmic antibody, rheumatoid factor and immunoglobulin (IgA, IgG, IgM) were all negative. Bronchoscopy was performed and the bronchoalveolar lavage fluid (BALF) culture was negative. Pathology of transbronchial lung biopsy of left lateral basal segment revealed that the pulmonary alveolar structure was intact, with fibrotic thickened alveolar septa, infiltrated by a few lymphocytes. Alveolar cavities were filled with a large amount of fibrinous exudation, which raised the possibility of interstitial pneumonia.

**Fig. 2:**
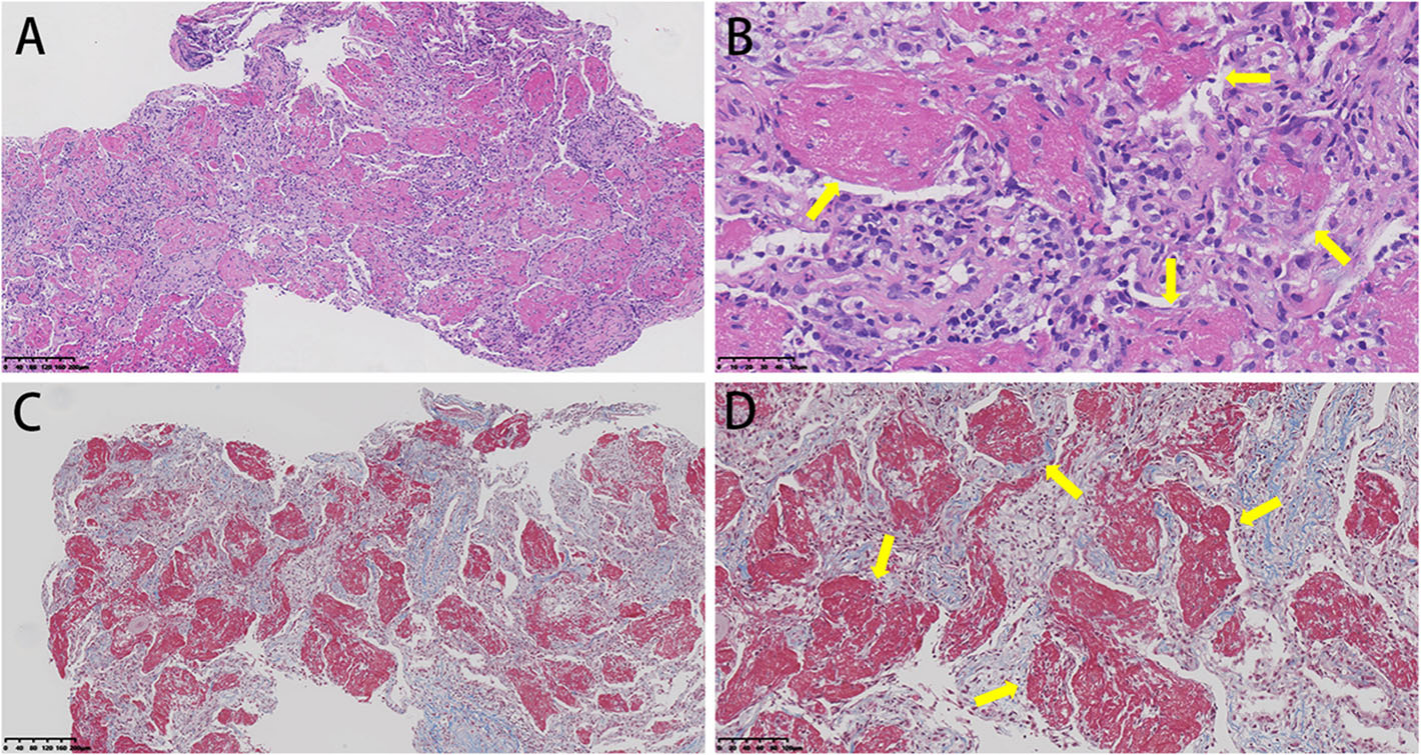
Histologic findings of case 1 on lung biopsy. Hematoxylin and eosin stain (**A**, ×100) (**B**, ×200) showed alveoli were filled with fibrinous exudate (arrows) without pulmonary hyaline membrane, the alveolar septum was thickened and infiltrated with a few lymphocytes, which were consistent with AFOP. Masson’s trichrome stain (**C**, ×100) (**D**, ×200) showed alveoli cavities were filled with fibrinous exudates (arrows)

On day 6th of admission, the patient had fever and chills again, with the highest temperature of 38.5 ℃. She was treated by intravenous moxifoxacin for 7 days, followed by imipenem-cilastatin, but she still had fever and developed tachypnea. On day 18th of admission, the chest CT showed that bilateral pulmonary lesions had progressed (Fig. 1B). Subsequently, Ultrasound-guided percutaneous lung biopsy was performed and histological examination demonstrated a large amount of fibrinous exudate in alveolar cavities, significantly widened alveolar septa, and a large number of lymphocytes, plasma cells and a few neutrophils. Organization foci composed of proliferative fibroblast was identified (Fig. 2). Combined with clinical details, the pathological finding raised possibility of AFOP. Acid fast stain, alcian blue stain, Grocott methenamine silver (GMS) and Periodic Acid-Schiff (PAS) stain were negative.

Intravenous corticosteroid (methylprednisolone, 80 mg/day for 3 days, followed by 60 mg/d for 1 week and 40 mg/day for another 1 week) was administered. Her clinical symptoms significantly improved and fever did not recur. At the 12th day of the steroid treatment, consolidations in both lungs reduced (Fig. 1C). The patient discharged on a regular tapering schedule of methylprednisolone with outpatient follow-up. Relapse occurred and follow-up CT showed new lesions in the left upper lobe after the dose was reduced to 12 mg around 9-month after discharge. She got improvement again after administration of methylprednisolone with the dose of 40 mg/day.

### Case 2

A 54-year-old non-smoking woman was admitted because of fever for 3 days. She had a history of systemic lupus erythematosus with secondary moderate anemia and had been treated with methylprednisolone and cyclophosphamide for 13 years.

**Fig. 3:**
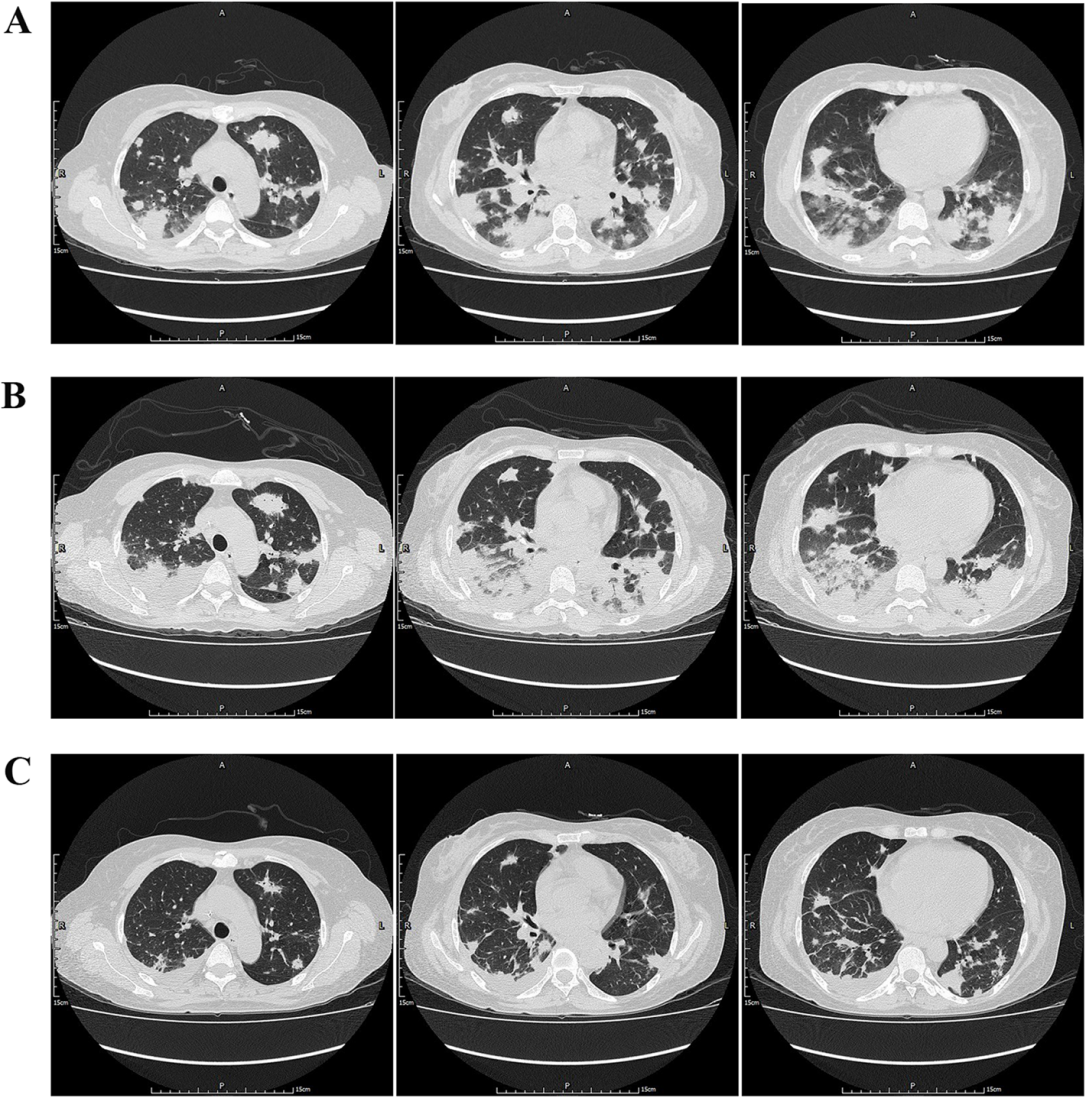
The initial CT images and the follow-up CT images of case 2. **A** Chest CT on admission revealed multiple nodules, consolidations and patchy opacities in both lungs. **B** CT images on day 14th of admission showed bilateral lesions increased and enlarged. **C** CT images at the 7th day of the steroid treatment showed absorption of bilateral lung lesions

Blood routine examination revealed elevated white blood cell count of 10.6*10^9/L with neutrophil predominance. Anti-cardiolipin antibody, anti-nuclear antibodies and anti-SSA antibody were positive while other serum examination for autoimmune disease including anti-neutrophil cytoplasmic antibody, rheumatoid factor and Coombs test were negative. Blood and sputum cultures were negative as well as serum pneumococcal antigen. Serum IgM for influenza A and B virus, adenovirus, respiratory syncytial virus, *Mycoplasma pneumoniae and Chlamydia pneumoniae* was negative, while interferon release test for tuberculosis was positive. Tumor markers including CEA, CA125, CA199, NSE and AFP were negative. Chest CT revealed multiple nodules, consolidations and opacities in both lungs, with slightly enlarged mediastinal lymph nodes (Fig. 3A).

Moxifoxacin was administered initially for anti-infective therapy and then switched to meropenem and linezolid on day 6th. Antituberculous and antifungal therapy were also added. However, the patient had recurrent high fever with temperature up to 40.5 ℃. The patient underwent bronchoscopy and transbronchial lung biopsy. The pathology demonstrated fragments of bronchial mucosa and fibrinous exudate. Alcian blue and PAS stain and culture of BALF were negative.

**Fig. 4:**
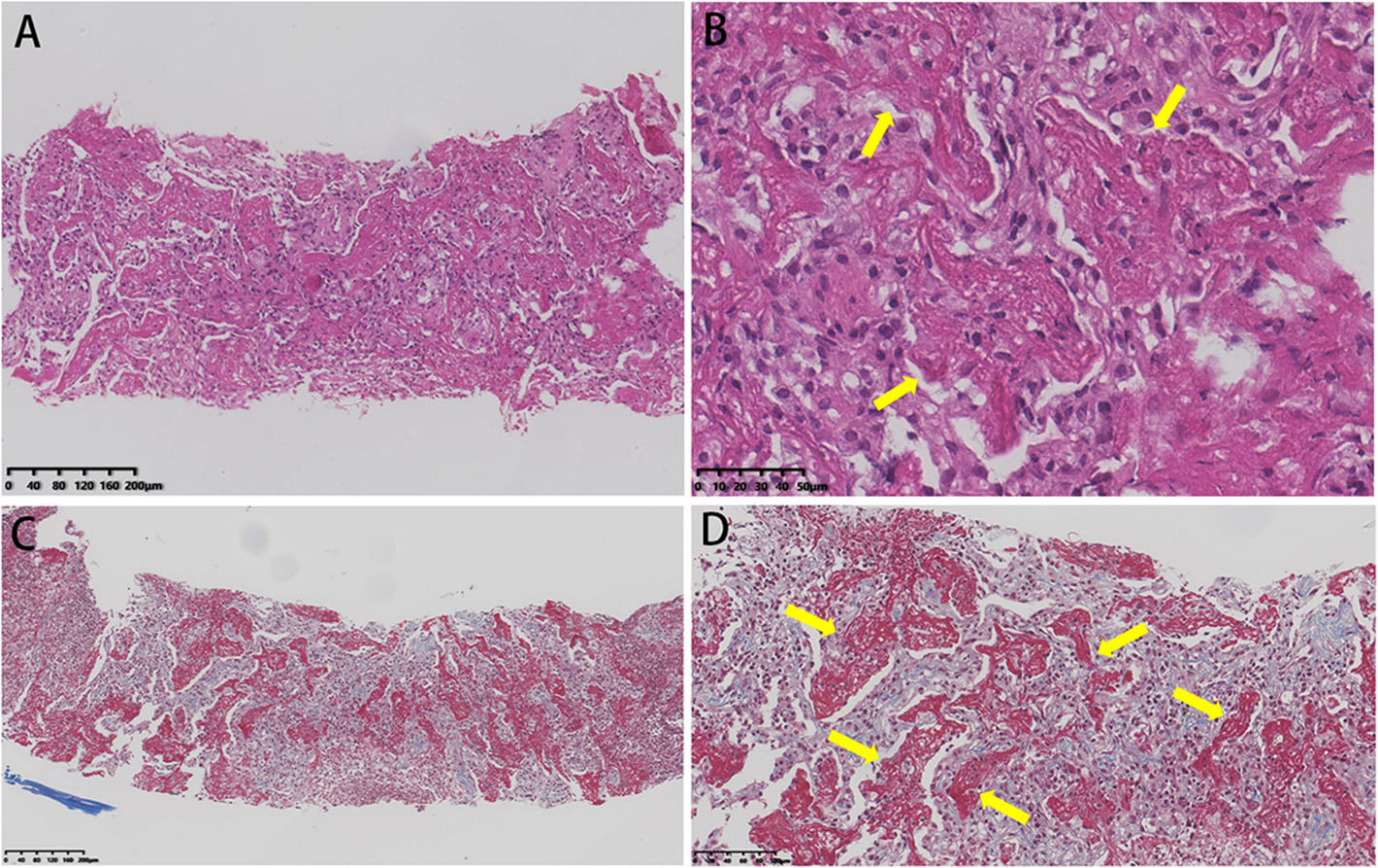
Histologic findings of case 2 on lung biopsy. Hematoxylin and eosin stain (**A**, ×100) (**B**, ×200) showed there was interstitial fibrosis and abundant of fibrous exudate filled in the alveoli(arrows), which was highlighted by Masson’s trichrome stain (**C**, ×100) (**D**, ×200) (arrows)

Two weeks after admission, chest CT indicated an increase of nodules and consolidations in both lungs with new development of a small amount of pleural effusion bilaterally (Fig. 3B). Ultrasound-guided percutaneous lung biopsy was performed and the pathology revealed that alveoli were filled with fibrinous exudate and the fibrotic thickened alveolar septa were infiltrated by large amount of lymphocytes (Fig. 4). GMS stain was negative. These findings were mostly consistent with AFOP. High dose of intravenous methylprednisolone (320 mg/d for 1 day and 200 mg/d for another 2 days, followed by 80 mg/day for 1 week) was administered intravenously, resulting in improvement of clinical symptoms and chest radiography findings (Fig. 3C).

## Discussion and conclusions

AFOP, first reported in 2002 in a case series involving 17 patients with acute respiratory failure [1], was defined as a subgroup of idiopathic interstitial lung disease [2] by the American Thoracic Society/European Respiratory Society statement in 2013. We performed a literature review of all reports of AFOP patients over the period from 2002 to 2019, by conducting a thorough search in PubMed and Web of Science databases. The search was limited to English publications. A total of 81 eligible reports [3–26] with non-overlapped 148 suitable cases were qualified for the literature review and a total of 150 candidates (including two patients in our study) were served as the study population for this analysis.

AFOPs have been reported mainly in USA, Europe and Asia. There was no clear gender difference and the mean age was 54.3±15.8 years (range, 38 days-84 years) (Table 1). Our two reported patients were 53 and 54 years old. Fifty cases were diagnosed as idiopathic AFOP, while the other 100 cases were considered secondary AFOP, which were associated with a wide range of medical conditions or risk factors (Table 2). It should be noted that there were two cases caused by immune checkpoint inhibitor (Nivolumab and pembrolizumab) [8, 21], which is one of the most effective treatments for metastatic melanoma and advanced non-small cell lung cancer in recent years. Smoking appeared not to be a risk factor.

**Table 1:** Clinical characteristic and prognosis of patients with AFOP

Variable	Total (*n*=150)	Idiopathic-AFOP (*n*=50)	Secondary-AFOP (*n*=100)
Age (y)	54.3±15.8	57.6±14.1	52.3±16.5
Gender, male^a^	65/128(51)	23/50(46)	42/78 (42)
Symptoms			
Fever	64(43)	27(54)	37(37)
Dyspnea	108(72)	40(80)	68(68)
Cough	106(71)	36(72)	70(70)
Chest pain	24(16)	14(28)	10(10)
Hemoptysis	9(6)	5(10)	4(4)
Progression, acute^a^	41/103(40)	16/41(39)	25/62(40)
Smoking status^a^	22/58(38)	8/28(29)	14/31(45)
CT pattern^a^			
Consolidation	79/145(54)	32/46(70)	47/99(47)
GGO	61/145(42)	14/46(30)	47/99(47)
Nodulars	29/145(20)	10/46(22)	19/99(19)
Mortality			
All-cause death	59 (39.3)	10 (20)	49 (49)
Related death	49 (32.7)	10 (20)	39(44)

Values are mean ± SD or n (%)

*AFOP *Acute fibrinous organizing pneumonia, *GGO *Ground-glass opacity

^a^not including all the cases for some were not reported

**Table.2:** Possible causes or associations with AFOP

Associations		n
Lung transplantation(LT)		25
Autoimmune diseases/CTD		13
	Juvenile dermatomyositis	1
	Systemic sclerosis	1
	Polymyositis	1
	Sjogren’s syndrome	2
	Anti-synthetase syndrome	2
	Systemic lupus erythematosus	2
	Collagen vascular disease	1
	CTD (with asbestos and fiberglass exposure)^a^	1
	Fibromyalgia	1
	Severe AA (suspected to be autoimmune)	1
Medications/Drugs		15
	Amiodarone (1 with zoologist exposure^a^)	2
	Abacavir	1
	Decitabine	2
	Bleomycin (1 with Aba infection^a^)	3
	Sirolimus	1
	Everolimus	1
	Nivolumab	1
	Cocaine	1
	Azacytidine	1
	Adjuvant chemotherapy&radiotherapy	1
	Pembrolizumab	1
Infection		14
	Lung abscess	1
	*Haemophilus influenza*	1
	Sepsis	1
	*Chlamydia* pneumoniae	1
	*Mycoplasm*a pneumoniae	1
	*Mycobacterium tuberculosis*	1
	Aspergillosis	1
	*Respiratory syncytial virus*	1
	Aba^a^	2
	*Pneumocystis jirovecii* (and HIV)^a^	1
	Influenza virus (after double LT)	1
	Not mentioned	2
Hematological malignances		9
	Lymphoma	5
	Acute leukemia	2
	Myelodysplastic syndrome	2
HSCT	(3 of lymphoma, 2 of leukemia)	5
Solid tumor		12
Environmental exposures		8
	Zoologist exposed to exotic animals(with usage of amiodarone )^a^	1
	Hair spay	1
	Coal miner	1
	Asbestoe and fiberglass exposure (with CTD)^a^	1
	Poultry	1
	Herbicide or pesticide	1
	Construction worker(with Aba infection )^a^	1
	Risk occupational exposure (not specified)	1
Whipple’s disease		1
Chronic glomerulonephritis		1
HIV	One with *Pneumocystis jirovecii* infection^a^	2

*AFOP *Acute fibrinous organizing pneumonia, *HSCT *Hematopoietic stem cell transplantation, *CTD *Connective tissue disease, *AA *aplastic anemia, *Aba* *Acinetobacter baumanii*

^a^the case was combined with another association

Clinical manifestations of AFOP were nonspecific. Most common symptoms were dyspnea, cough (87 had a non-productive cough) and fever (Table 1). Other symptoms included hemoptysis, fatigue, chills, night sweat and weight loss. One patient was asymptomatic. Fever was considered to be more common in AFOP when compared with other forms of interstitial pneumonia [27]. The disease progression was acute in 41 patients, subacute in 62 patients, and not specific in the rest.

The most common CT findings were bilateral diffuse patchy consolidations, often with basal or peripheral dominance. There were 116 cases showing bilateral involvement. The most common imaging pattern was consolidation in 79 patients, of which 28 cases also demonstrated ground-glass opacity(GGO); other common patterns were GGO in 61 patients and nodules in 29 cases (Table 1). Consolidation was more frequently demonstrated in idiopathic-AFOP patients, but conversely, GGO was more frequently demonstrated in secondary-AFOP patients. Pleural involvement was uncommon, with mild bilateral effusion in 7 cases, right pleural effusion in 3 patients and pneumothorax in 2 patients. In addition, CT scan showed interstitial pneumonia in 11 cases and a solitary mass in 2 cases. All of these atypical findings were exclusively seen in the secondary-AFOP.

The nonspecific clinical features frequently resulted in misdiagnosis and a delay in the diagnosis of AFOP. At the time of initial diagnosis, most cases were misdiagnosed as lung infection, while a few cases were misdiagnosed as lung cancer. Beasley and his colleagues [1] described a mean time from onset of symptoms to diagnosis of 19 days, and Gomes et al. [4] reported a mean time of 43.9 days.

The diagnosis of AFOP depends on the pathology of a lung biopsy specimen. The lung lesion tissue was obtained by surgical lung biopsy (mainly by video-assisted thoracic surgery) in 60 patients, transbronchial lung biopsy in 43 patients, image-guided (CT- or ultrasound-guided) percutaneous lung biopsy in 31 patients, autopsy in 13 patients and unspecified in 3 patients. BALF was performed in 28 patients before pathological diagnosis and no conclusive pathology were found. For both of our cases, either BALF culture or transbronchial biopsy was conclusive. To confirm the diagnosis, a second lung biopsy (percutaneous lung biopsy) was performed in our two cases and the pathology was consistent with AFOP.

The main pathological features of AFOP are intra- alveolar fibrin deposition in the form of fibrin “balls” and patchy organized pneumonia [1]. Its minor features are acute or chronic inflammation and type II pneumocyte hyperplasia adjacent to areas of fibrin deposition, alveolar septal expansion with myxoid degeneration, and only minimal changes in the pulmonary tissue areas without fibrinous exudation. The pathological differential diagnoses include bronchiolitis obliterans organizing pneumonia(OP), eosinophil pneumonia(EP), and diffuse alveolar damage(DAD) [1]. Fibrin deposits could appear in both the DAD and OP patterns, but they are not a predominant histologic finding, and do not show the intra-alveolar fibrin ball pattern. The AFOP further differs from DAD in that classic hyaline membranes are absent, and differs from EP by the lack of prominent eosinophils [1]. Surgery biopsy is the best for tissue sampling to make AFOP diagnosis, as it can minimize the missing area for hyaline membranes in DAD. However, we suggest that the method of lung biopsy should be based on the location of lung lesions and patients tolerance to the operations.

Regarding treatment for AFOP, the most common therapy was corticosteroids, in 132 of 150 patients (88 %). There is no consensus on the dose and duration of corticosteroids. Twelve patients were administered with a pulse therapy of corticosteroids (prednisone 500 mg/day to methylprednisolone 1000 mg/day), and 4 died (one of them from other complications). Relapse may occur during steroid tapers and symptoms mostly could relieve again after higher doses were resumed. Immunosuppressive agents such as cyclophosphamide, mycophenolate mofetil, cyclosporine, azathioprine, tacrolimus etc. had been tried in 17 patients, all combined with corticosteroids, with varying results. AFOPs secondary to autoimmune diseases may benefit most from immunosuppressant. Other reported effective drugs were antibiotics (especially for those caused by infection), etanercept [25], indomethacin [28] and immunoglobulin [23].

Discontinuation of relevant drugs is of critical important. In 13 drug-related AFOP patients, 9 candidates completely recovered by drug discontinuation with corticosteroids. However, two patients died and another two died of other complication. Partial lobectomy/lesion resection was reported as a successful cure when the disease was relatively localized [29]. Hematopoietic stem cell transplantation [30] or lung transplantation [6, 30, 31] might be an option to treat AFOP which was refractory to medical treatment. However, further studies are required to follow up for possible recurrence of AFOP.

Among 150 reported cases, 59 (39 %) patients died with 10 cases being unrelated to AFOP) (Table 1). AFOP after lung transplantation seemed to have relatively poor prognosis [26]. Patients with acute-onset or secondary-AFOP had higher mortality rate than their corresponding cases. Furthermore, we discovered that AFOP with development of dyspnea, GGO in CT scan and lack of consolidation seemed to be associated with poor prognosis. However, further evaluation is required to confirm this finding.

In conclusion, we performed the so far most comprehensive review regarding AFOP, including 150 patients over an 18-year period. Given the various etiologies, nonspecific clinical presentations and insufficient understanding of pathology, AFOP probably had been under diagnosis or misdiagnosed. Our two cases highlight the importance of being aware of this uncommon pattern of acute lung injury. AFOP should be considered one of differentials in suspected pulmonary infection cases unresponsive to empirical antibiotic therapy, especially with CT patterns of basal or peripheral-predominant consolidation, with or without GGO or nodular opacities. Dedicated tissue sampling and thorough pathologic evaluation are essential for diagnosis and therapeutic guidance and hence to yield satisfactory outcome. While corticosteroids was recommended as the most effective therapy, other treatment options should be considered depending on the etiology and disease severity.

## Data Availability

The datasets used and analysed during the current study are available from the corresponding author on reasonable request.

## Abbreviations

AFOP: Acute fibrinous and organizing pneumonia
BALF: Bronchoalveolar lavage fluid
CTD: Connective tissue disease
DAD: Diffuse alveolar damage
EP: Eosinophil pneumonia
GGO: Ground-glass opacity
GMS: Grocott methenamine silver
OP: Organizing pneumonia
PAS: Periodic Acid-Schiff
